# Characterization of CO_2_ Adsorption Behavior in Pyrolyzed Shales for Enhanced Sequestration Applications

**DOI:** 10.3390/molecules30214196

**Published:** 2025-10-27

**Authors:** Asmau Iyabo Balogun, Haylay Tsegab Gebretsadik, Jemilat Yetunde Yusuf, Hassan Soleimani, Eswaran Padmanabhan, Abdullateef Oluwagbemiga Balogun

**Affiliations:** 1 Southeast Asia Clastic and Carbonate Research Laboratory (SEACARL), Institute of Sustainable Energy (ISE), Universiti Teknologi PETRONAS, Bandar Seri Iskandar 32610, Perak, Malaysia; haylay.tsegab@utp.edu.my; 2 Department of Geoscience, Universiti Teknologi PETRONAS, Bandar Seri Iskandar 32610, Perak, Malaysiaeswaranpadma@yahoo.com (E.P.); 3 Department of Fundamental and Applied Sciences, Universiti Teknologi PETRONAS, Bandar Seri Iskandar 32610, Perak, Malaysia; jemilat_19001626@utp.edu.my; 4 Department of Computing, Universiti Teknologi PETRONAS, Bandar Seri Iskandar 32610, Perak, Malaysia; abdullateef.ob@utp.edu.my

**Keywords:** CO_2_ capture, kinetics, pyrolysis, spent shale, adsorption, temperature-programmed desorption (TPD)

## Abstract

Mitigating climate change through the reduction of atmospheric CO_2_ emissions remains a critical global priority. Solid adsorbents, particularly shales, have become promising options for CO_2_ storage due to their favorable structural and chemical properties. In this study, a solid sorbent was developed by pyrolyzing shale at 800 °C under a nitrogen (N_2_) atmospheric condition, yielding spent shale. The key physicochemical properties influencing CO_2_ sorption were characterized using X-ray diffraction (XRD), Field Emission Scanning Electron Microscopy (FESEM), Brunauer–Emmett–Teller (BET) surface area analysis, and Temperature-Programmed Desorption (TPD). Mineralogical analysis revealed the presence of quartz, feldspars, clays, and carbonate minerals. The spent shale exhibited surface areas of 30–34 m^2^/g and pore diameters ranging from 3 to 10 nm. TPD results confirmed the presence of active adsorption sites, with a maximum CO_2_ sorption capacity of about 1.62 mmol/g—surpassing several commercial sorbents. Adsorption behavior was best described by the Sips and Toth isotherm models (R^2^ > 0.99), indicating multilayer and heterogeneous adsorption processes. Kinetic modeling using both pseudo-first-order and pseudo-second-order equations revealed that CO_2_ uptake was governed by both diffusion and chemisorption mechanisms. These findings positioned spent shale as a low-cost, efficient sorbent for CO_2_ storage, promoting circular resource utilization and advancing sustainable carbon management strategies. This novel shale-derived material offers a competitive pathway for carbon capture, storage, and sequestration applications.

## 1. Introduction

Carbon dioxide (CO_2_) stands out as the most prevalent greenhouse gas (GHG) acknowledged for its pivotal role in driving climate change and global warming [1]. Various nations, including the USA, China, and Malaysia, are currently advocating for the reduction in GHG emissions to protect the global climate. In January 2022, atmospheric carbon dioxide levels reached a peak of 418.19 ppm, a significant increase from 403 ppm in 2016 [2]. The increase in the rate of CO_2_ emission to the environment as a result of the combustion of fossil fuels (due to the high percentage of CO_2_ present in shale) has raised more concern about global climate change as well [3]. In response to this critical issue, it is imperative to implement effective strategies and develop innovative, cost-efficient technologies suitable for the CO_2_’s sequestration and utilization. The adoption of CCUS (Carbon Capture, Utilization, and Storage) techniques has become widespread. CCUS involves injecting CO_2_ into stable geological formations, such as shale, using predominantly sorption techniques to ensure its permanent retention in the geological formation [4].

The sorption of CO_2_ in shale holds significant promise for substantially reducing costs, enhancing safety, and prolonging the storage of CO_2_ for extended periods [5]. In addition, the low permeability, low porosity, and some of the minerals present in shale can adsorb large amounts of ions, water, natural gas, or other substances, making it an effective adsorbent material [6,7,8]. In the last few decades, several studies have shown that Shale formation possesses a significant storage capacity for CO_2_ under simulated conditions, and this has helped in minimizing the storage cost, which makes it an economically viable option compared to other solid adsorbents [9,10]. Zardari et al. [11] confirmed that CO_2_ sorption in shale occurs through both physical and chemical adsorption mechanisms. In physical sorption, van der Waals forces attract some gas molecules to the shale surface [12,13]. The CO_2_-quadrupole moment also aids its strong interaction with the electric field gradient of shale, hence, enhancing its adsorption as CO_2_ has the highest quadrupole moment among various gases, followed by CO (carbon monoxide), N_2_ (nitrogen), H_2_ (hydrogen), and CH_4_ (methane) [14].

However, several reports have enlightened the utilization of spent shales as solid sorbents in the last few years [15,16] based on their petrochemical characteristics. Spent shales, on the other hand, are end-products or solid residues of oil shale obtained through the oil-shale retorting process. These solid residues are quite available because they are usually discarded or transported back to the milling site, where they are dumped [17], hence causing more environmental pollution. Furthermore, the sorption potential of spent shales, as reports have shown that spent shales possess a larger surface area than raw shale, including a more microporous nature that enables the capturing of CO_2_ molecules, and retention of sorption-supporting minerals, to mention a few [18,19]. These properties are the prominent characteristics of a sustainable sorbent, especially for CO_2_ storage. Wang et al. [20] stated that the surface area and pore structure formed within the spent shale during pyrolysis have a major impact on its reaction with gas as a solid sorbent, while Wang, et al. [21] conducted an adsorption measurement on pyrolyzed coals (semicoke) and discovered that the surface area, pore diameters, as well as adsorption potential of these semicokes increased effectively with pyrolytic temperature during the pyrolysis process. Furthermore, Han et al. [17] summarized the potentiality of spent shale as a sorbent material based on the nature and characteristics of its pore structures, with a significant outcome, and Bai et al. [22] reported that the internal structures of spent shale can affect the efficient separation of gases during a sorption process, which will result in adequate recovery of pure gases. Alaloul et al. [23] performed a comprehensive review and confirmed that oil shale waste can be utilized for different purposes, and Bayaidah et al. [24] utilized spent shale in concrete production and stated that the chemical composition of spent shale aids its utilization for this purpose.

Thus far, most of these studies have shown that there has been more focus on the surface characteristics of spent shale and its utilization for other purposes, different from CO_2_ sorption. Invariably, there is a gap in knowledge on the CO_2_ sorption capacity of spent shale, which includes the effect that the pyrolytic temperatures can have on the sorption capacity of the spent shale or the influence of the pore variation after the pyrolysis on its sorption performance. Hence, understanding their geological origin, processing pathways, and physicochemical characteristics is essential for assessing their suitability as potential solid sorbents.

Therefore, this study aims to investigate the sorption capacity of some spent shales, obtained from hydrocarbon-bearing Marcellus shale, as effective solid sorbents for CO_2_ storage. Marcellus shales were employed as reports have proved that after their hydrocarbon production, numerous pores are available in the spent shales generated, which can serve as CO_2_ storage sites [25,26]. Thus, exploring their potential as an alternative to commercially available sorbents. In so doing, the physicochemical characteristics and the CO_2_ sorption analyses were investigated. The CO_2_ adsorption experiments were performed at 8 MPa to replicate subsurface reservoir conditions typical of geological storage (5–10 MPa), rather than atmospheric post-combustion capture. This pressure range reflects realistic conditions encountered in deep formations and allows a more accurate assessment of CO_2_ -geological formations interactions for long-term storage applications [27]. To validate the results obtained, some isotherm and kinetic models were employed. Finally, the results were compared with the raw shale adsorption behavior.

The significance of this research lies in its contribution to sustainable development by providing an alternative use for spent shales, thus reducing waste and promoting resource efficiency (supporting United Nations Sustainable Development Goal (UN-SDG): 12 Responsible Consumption and Clean Energy).

## 2. Results and Discussion

### 2.1. Morphology Characterization

#### 2.1.1. Pore Distribution

Understanding the relationship between porous structures and adsorption aids in the calculation of gas in situ; thus, the characteristics of pores, such as pore shapes and distribution of pore sizes, help in determining the interconnectivity between pores and the sorption behaviors of shale.

Surface areas are commonly reported as BET surface areas obtained by applying the theory of Brunauer, Emmett, and Teller to nitrogen adsorption isotherms measured at 77 K, as nitrogen at 77 K is considered to be a standard adsorbate for surface area and pore size analysis. This is a standard procedure that allows for comparisons among different materials and with benchmark materials from the literature [28]. The pyrolyzed samples, S3-PY and S6-PY, possess larger surface area, pore sizes, and pore volume than the samples S3 and S6, as shown in Table 1. A notable increase in the pore size of S3-PY was observed compared to S3. This could be attributed to the decomposition of kerogen because kerogen holds a significant amount of micropores in shale, as reported by Gonciaruk et al. [29]. Thermal stress and kerogen pyrolysis can result in the development of more mesopores, and more pores and fractures can connect to form micro-fractures [30,31]. The kerogen type of sample S3 may have easily pyrolyzed during the pyrolysis process, thereby making S3-PY have more extensive pore merging, leading to a higher average pore size compared to the others. It can also be observed that the quantity of N_2_ adsorbed by the pyrolyzed samples is greater than the amount adsorbed by the non-pyrolyzed samples. The increased N_2_ uptake after pyrolysis indicates that the shale adsorption capacity is strongly related to the factors that can produce numerous pores [28]. The presence of abundant mesopores and micropores and a larger surface area in adsorbents results in a greater interactive energy between adsorbate and adsorbent. Therefore, the types of pores and surface areas can result in increased adsorption performance.

**Figure 1:**
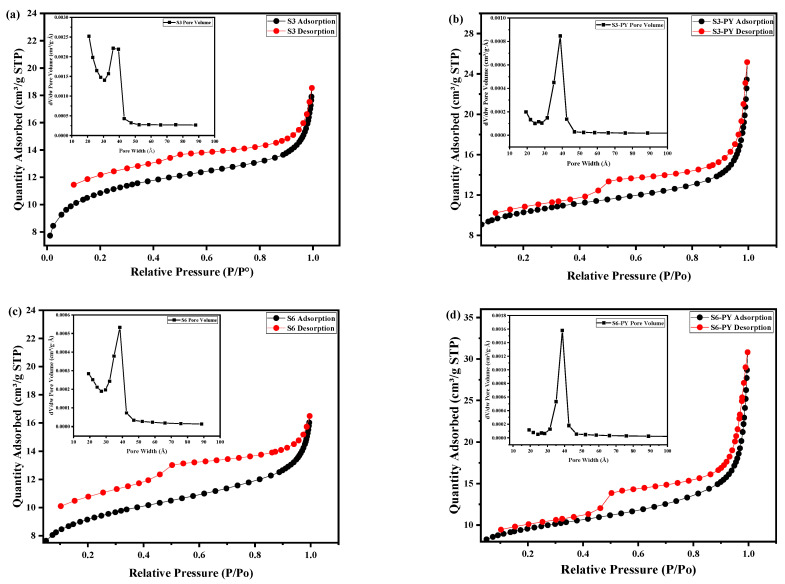

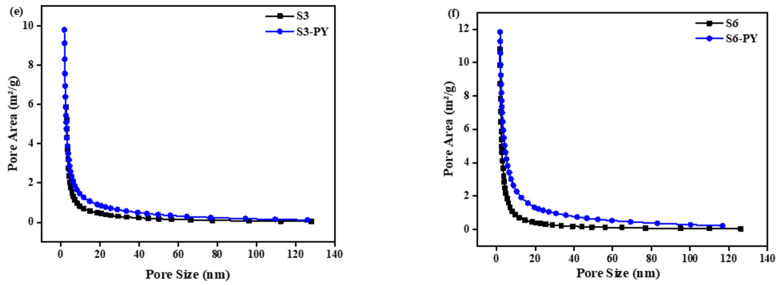
N_2_ adsorption/desorption curves and the corresponding pore size distributions (insets) of (**a**) S3, (**b**) S3-PY, (**c**) S6, (**d**) S6-PY; and BJH pore size distribution of (**e**) S3/S3-PY, (**f**) S6/S6-PY.

The isotherm curves for the studied samples were described as Type II isotherm curves with an H2 hysteresis loop, as shown in Figure 1a–d. This description is in accordance with the IUPAC and BET classification. The Type II isotherm curves revealed complete monolayer adsorption up to the relative pressure of approximately 0.4 before multilayer adsorption occurs. Neck-like and wide-body pores or ink bottle-like pores obtained from the hysteresis loop confirmed the meso-macroporous nature of the samples. These features have been reported to be favorable for CO_2_ sequestration and storage, as the size of the pores present on the samples can adsorb more CO_2_ (33 nm) physically [32].

The steepness of the capillary condensation steps suggested the presence of mesopores, which was further confirmed by the pore size distribution in the inset of Figure 1a–d.

The insets of Figure 1a–d show the pore volume distribution of the samples in relation to the pore width. Samples S3, S3PY, S6, and S6-PY exhibit pore width distribution in the range of 15–50 Å (1.5–5 nm), indicating the inner width of the pores. In the raw S3 and pyrolyzed samples, a pore filling was observed between 29 and 47 Å (2.9–4.7 nm) with a relatively lower peak pore volume, whereas S3-PY and S6-PY samples displayed a pore filling at 31–46 Å (3.1–4.6 nm), reflecting a more uniform pore structure. These apparent peaks observed around 3–4 nm in the inset graph of the pore volume of all samples show an artifact associated with cavitation and pore-blocking effects during desorption. The pore volume significantly increased in the pyrolysed samples as a result of a greater concentration of uniform pore size. Thus, pyrolysis of shale samples results in the development of a uniform pore network, concentrating on mesopores, which would contribute significantly to enhanced adsorption capability. The BJH pore size distribution analysis, as shown in Figure 1e,f, reveals the porosity in both raw and pyrolyzed samples, with the pyrolyzed samples having a larger area of pores. It further shows that the mesopore development becomes dominant after pyrolysis, reflecting structural reorganization and mineral decomposition at elevated temperature [33].

#### 2.1.2. Mineral Contents

**Figure 2:**
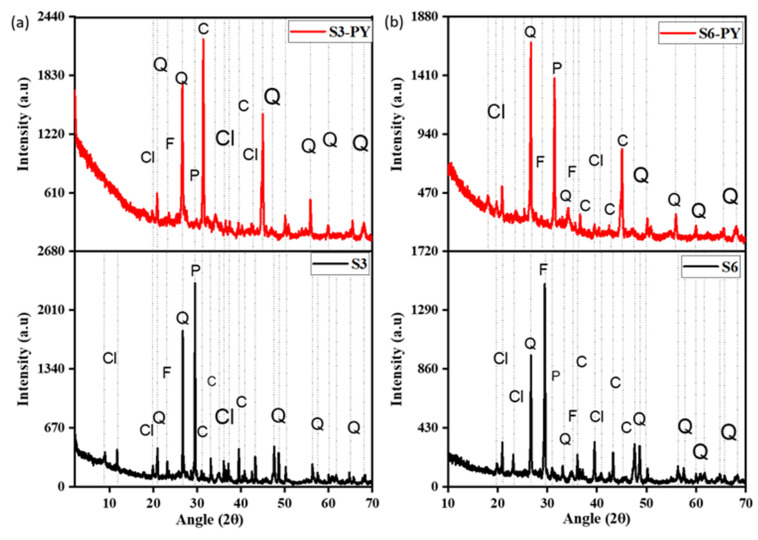
The mineralogical content of (**a**) S3 and (**b**) S6 before and after pyrolysis, Cl = clay, Q = quartz, F = feldspar, P = pyrite, and C = carbonate minerals, respectively.

The XRD spectra of the shale samples S3, S6, S3-PY, and S6-PY indicate the presence of silicate minerals, dominantly quartz, feldspar, clay minerals, and carbonates, as shown in Figure 2. The intensity of the clay minerals present in the pyrolyzed samples reduces due to the heat of pyrolysis, eliminating some of their absorbed water. The presence of minerals such as quartz, feldspar, clay minerals, and carbonates in the samples influences the sorption capacity of shale due to the CEC (cation exchange capacity) of some of the minerals, specifically the clay minerals, as well as the inner sphere complexation that aids a direct bonding of the molecules to the surface of the minerals during chemical sorption [10]. Likewise, calcites are known to have a high affinity for CO_2,_ with the molecular size of CO_2_ occupying several spaces, and also the energy of Ca-O, C-C, C-O, and O-O pair interaction of Lennard-Jones ranging from 0 to 1.43 × 10^−2^, 3.08 × 10^−4,^ and 5.21 × 10^−5^ KJnm^6^mol^−1^, respectively, indicates an easier bond of CO_2_ on oxygen bonded with calcium molecules in Calcite [34].

**Figure 3:**
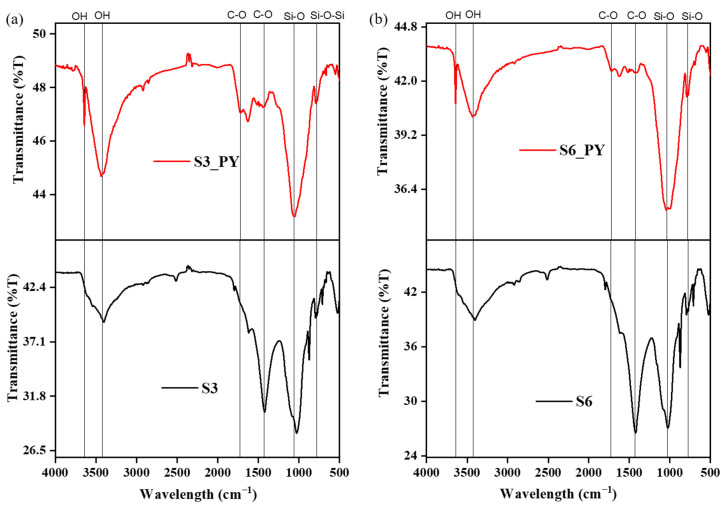
The variation in the functional groups of (**a**) S3 and (**b**) S6 before and after pyrolysis.

Furthermore, the abundance of feldspar and quartz in all samples reflects their stability at higher temperatures after the pyrolysis process, and these minerals have also been reported to influence the sorption behavior of shales [35,36,37]. The reduction in the peaks of most clay minerals is due to dehydroxylation at about 100 °C and deformation above 450–500 °C, e.g., kaolinite. However, some clay minerals, such as illite, deform at temperatures greater than 900 °C, and the internal structures of montmorillonite, kaolinite, and illite play a vital role in the sorption of CO_2_ on shales [9,38]. In addition to the mineral composition, the FTIR spectra indicate the presence of O-H, Si-O, and C-O functional groups in both raw and spent shales, as shown in Figure 3.

These functional groups confirm the presence of the silicate and carbonate minerals obtained from the XRD, as some silicate minerals, such as kaolinite, illite, and other clay minerals, possess some absorbed moisture, which brought about the O-H group at 3000–3600 cm^−1^ [39,40]. Also, some silicate minerals such as feldspar and quartz with Si-O bonds appear at about 400–1100 cm^−1^ [41], and some carbonate bonds appear at 700–1400 cm^−1^ [42]. Reports have shown that these functional groups play vital roles in the adsorption behavior of any samples they are found in, as they can adsorb or desorb ions and molecules physically or chemically [43,44].

#### 2.1.3. Surface Morphology

**Figure 4:**
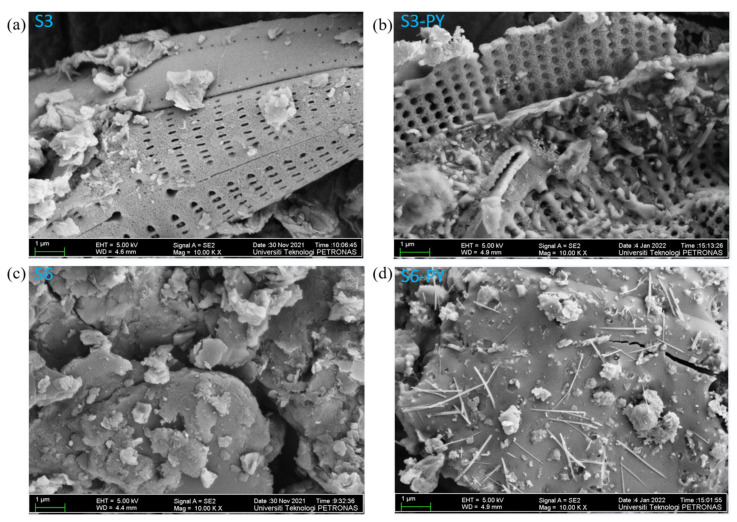
FESEM images of (**a**) S3, (**b**) S3-PY, (**c**) S6, (**d**) S6-PY at 1 μm resolution and 10k magnification ratio.

A comparison of FESEM images obtained before and after pyrolysis revealed significant changes in the pore morphology of the studied sample. Before pyrolysis, the images of the raw samples (Figure 4a,c) show the presence of various pore sizes due to the low porosity of an undisturbed sample. The pyrolysis of the samples created a pore network that increases the porosity of the samples due to the release of moisture and volatiles at elevated temperatures (Figure 4b,d).

In addition, the pyrolyzed samples reveal the presence of both intra-and intergranular pore structures, which are preserved in the mineral particles of feldspar and quartz, and also intercrystalline pores associated with the space generated in the clay minerals during dehydroxylation. Figure 4a also reveals the striae formation of silica with visible nanopores in sample S3. The distinct pore size, as well as the number of pores, increased in samples S3-PY (Figure 4b) due to the pyrolysis process. Furthermore, illite fibers were observed on the authigenic quartz and feldspar in sample S6-PY (Figure 4d), aligning with the sheet-like and hexagonal structures of illite identified in sample S6 (Figure 4c). The weight percent (wt.%) of each elemental composite in the studied samples was measured through the FESEM-EDX, as illustrated in Table 2.

**Figure 5:**
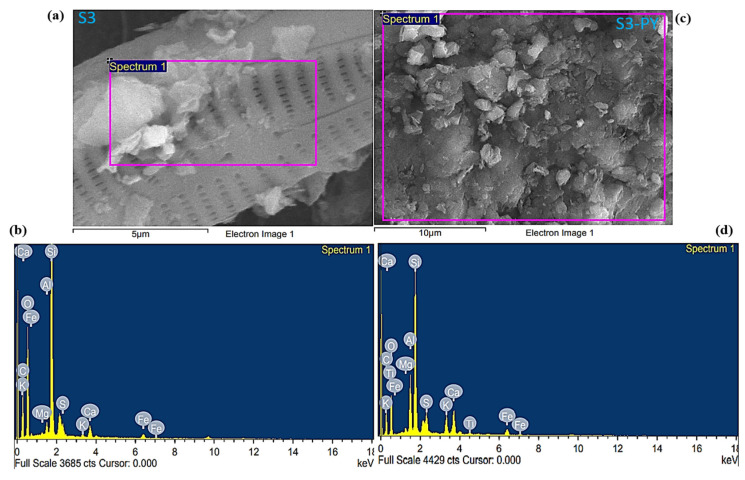
FESEM-EDX of (**a**,**b**) S3 (**c**,**d**) S3-PY.

**Figure 6:**
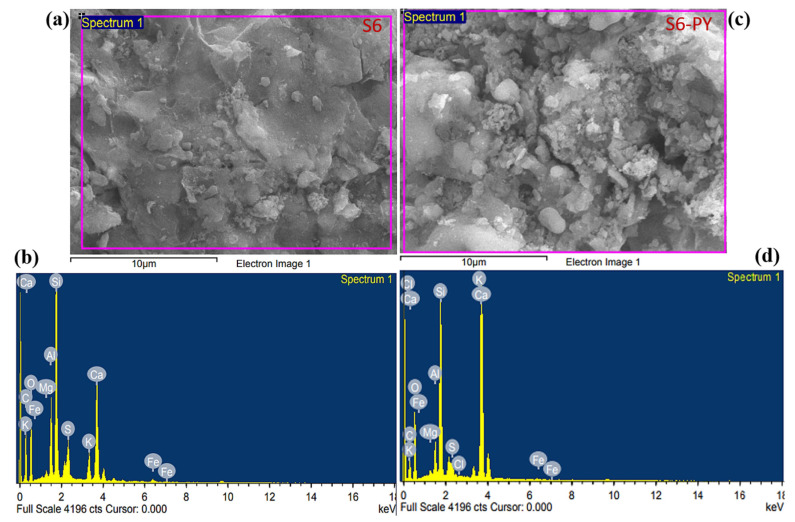
FESEM-EDX of (**a**,**b**) S6 (**c**,**d**) S6-PY.

Based on the EDX analysis, all samples contain a large amount of carbon, oxygen, silicon, and calcium, with some amount of aluminum, magnesium, potassium, iron, and sulfur, which are the main elements found in the silicate and carbonate minerals, as shown in Figure 5 and Figure 6. The carbon content of S3-PY is nearly identical to that of S3, indicating minimal change upon pyrolysis. In contrast, S6 exhibits a higher carbon content than S6-PY, and the observed decrease in carbon content after pyrolysis can be attributed to the loss of hydrocarbons present in the shale oil [45]. The amount of calcium in the pyrolyzed samples increased, which describes the abundance of calcite after the pyrolysis, as it is one of the minerals that are stable at higher temperatures [46]. Although these elements confirm the presence of the minerals observed from the XRD but the variation in the wt.% before and after pyrolysis is based on the thermal stability of each mineral [47]. In addition, the FESEM micrographs reveal some pieces of evidence that support the pore structures and mineralogy of the studied samples. Thus, they are good indications that the samples are porous and have significant sites for gas sorption.

### 2.2. CO_2_ Sorption Measurements

**Figure 7:**
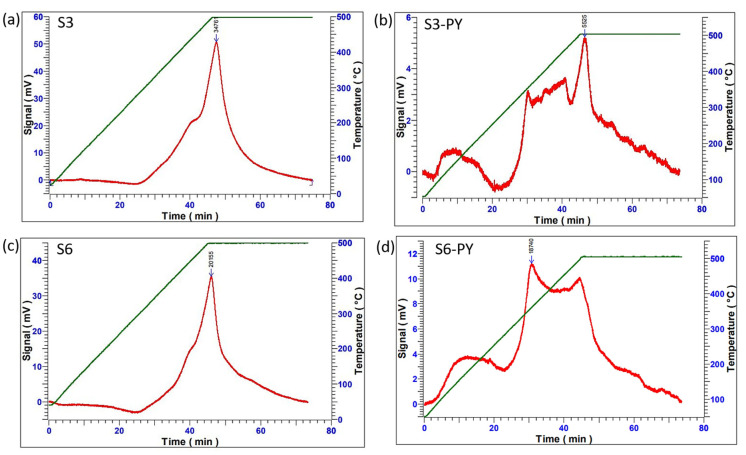
The CO_2_ sorption/desorption process on (**a**) S3, (**b**) S3-PY, (**c**) S6, (**d**) S6-PY.

The CO_2_ sorption capacity of both raw and pyrolyzed samples was measured through the Temperature Programmed Desorption (TPD) analysis and volumetric technique. The TPD techniques involve measuring the acidic/basic strength of the spent shale for sorption performance. This technique was employed primarily to probe the nature of CO_2_- shale surface interactions and to assess the strength of the adsorption sites. The procedure involves heating the samples under N_2_ gas at 28 °C, followed by the injection of CO_2_ until the temperature reaches 75 °C, and is held for 30 min to obtain the sorption measurement, then being completed by injecting Helium gas up to 500 °C for the next 30 min to desorb any form of chemisorbed CO_2_. The results showed that all samples obtained their maximum desorption peaks at around 450–500 °C (i.e., the highest temperature at which the chemically adsorbed CO_2_ in the spent shale completely desorbs [48,49]. As shown in Figure 7, the desorption spectrum (red line) indicates how the CO_2_ is being desorbed with increasing temperature until it attains its maximum peak, where it begins to fall. This same point aligns with the maximum temperature for chemisorbed CO_2_ (green line), which indicates that the temperature required for the desorption of CO_2_ was quite high as a result of the higher interaction of the CO_2_ with the active sites, thereby making it more difficult to desorb [49]. Invariably, this determines the bond strength between them as chemisorption, which can be attributed primarily to the presence of reactive clay mineral surfaces and carbonate decomposition products. Figure 7 shows that all studied samples have great adsorption sites for CO_2,_ as well as a great affinity for CO_2,_ and support the chemisorption process.

**Figure 8:**
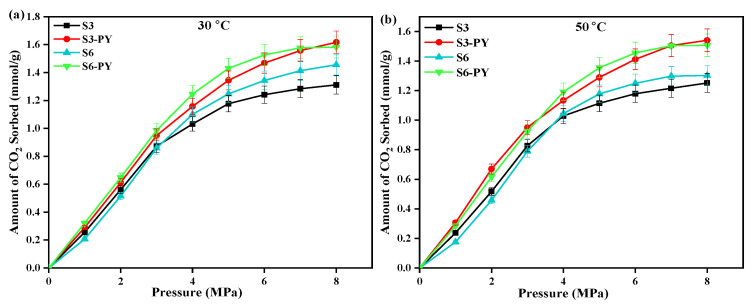
Amount of CO_2_ sorbed and error bar points on raw and pyrolyzed samples S3 and S6 at (**a**) 30 °C, (**b**) 50 °C, and up to 8 MPa pressure.

In addition to the TPD analysis, the results obtained through the volumetric technique showed that the sorption of CO_2_ on all studied samples increases relatively with pressure at an isothermal condition (See Figure 8). Also, the amount of CO_2_ sorbed varied with samples as the maximum amount of CO_2_ sorbed on raw samples S3 and S6 is 1.31 mmol/g and 1.45 mmol/g, while the pyrolyzed samples S3-PY and S6-PY obtained 1.62 mmol/g and 1.58 mmol/g, respectively (See Figure 8a). This indicates that the amount of CO_2_ adsorbed increased after pyrolysis. Furthermore, the results obtained from the volumetric techniques showed that the studied samples followed the same pattern under the same operating conditions, which indicates that the pyrolyzed shale samples also possess the force of attraction to sorb the CO_2_ molecules, similar to the raw shale samples. In addition, the persistence and transformation of clay minerals due to the pyrolysis treatment directly influence surface area and adsorption sites, which correlate with the observed uptake values of the pyrolyzed samples under the same operating conditions. In addition, the coexistence of mesopores and micropores is complementary because the micropores aid the sorption capacity by providing strong sites, while the mesopores improve gas transport and diffusion, ensuring that sorption sites remain accessible under high-pressure conditions. These explain why the spent shale exhibits high sorption capacity. Therefore, under relative geological conditions, spent shales showed effective sorption performance, making them suitable for industrial applications.

### 2.3. Sorption Equilibrium Modeling

**Figure 9:**
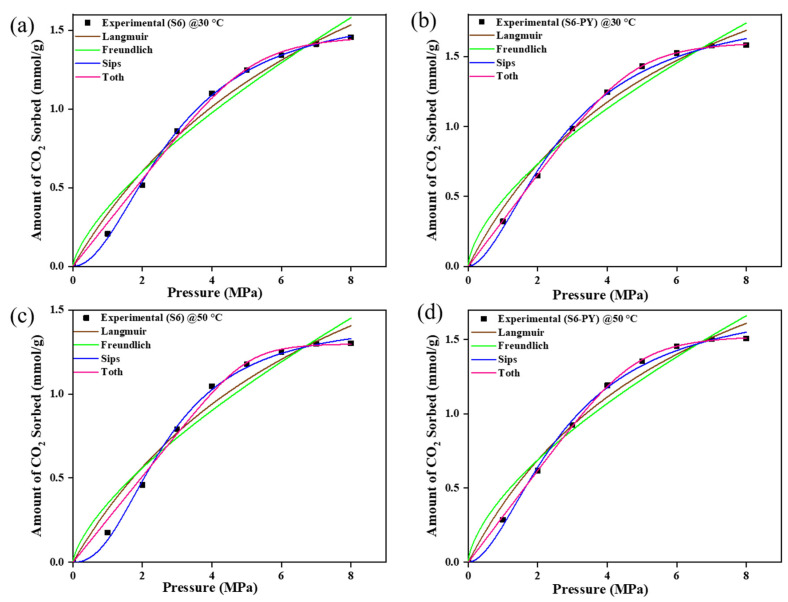
The Isotherm curve-fit of samples S3 and S3-PY data at 30 °C (**a**,**b**) and 50 °C (**c**,**d**).

**Figure 10:**
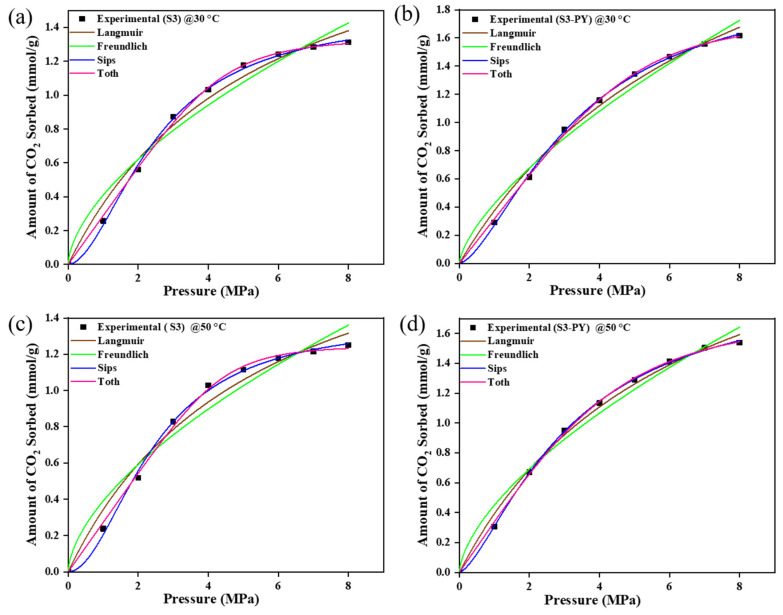
The Isotherm curve-fit of samples S6 and S6-PY data at 30 °C (**a**,**b**) and 50 °C (**c**,**d**).

The CO_2_ sorption experiments for raw and pyrolyzed samples S3 and S6 were modulated with Langmuir, Freundlich, Sips, and Toth models as shown in Figure 9 and Figure 10. The values of each parameter obtained from the plots are illustrated in Table 3 and Table 4. The estimated values/parameters given in Table 3, Table 4, Table 5 and Table 6 were obtained using a non-linear regression analysis with 95% confidence intervals (CI). The experimental data showed a good match with all models; i.e., the sorption behaviors of all studied samples are best fitted with the Freundlich, Langmuir, Sips, and Toth models, with their R-squared value greater than 0.96.

However, the pyrolysed samples showed greater agreement with the Sips and Toth model (R^2^ > 0.99), with the n-values of Sips exceeding 1 and being less than 1 for the Toth model.

The equilibrium models showed that the sorption process is supported by a heterogeneous phenomenon, as they conformed best to both the Sips and Toth models. This is because the Sips and Toth models best define a heterogeneous adsorption whereby the molecules of the adsorbate disperse throughout the surface of the adsorbent.

For the Sips model, the n-values were consistently greater than 1, indicating a heterogeneous adsorption surface with consistent adsorption behavior and the presence of adsorption sites created by pyrolysis. In contrast, the Toth model yielded n-values less than 1, suggesting a broader distribution of adsorption energies and highlighting the non-ideal, heterogeneous nature of the surface after pyrolysis. These results collectively imply that the structural modifications induced by pyrolysis enhance the heterogeneity of pore formation, thereby improving the samples’ capacity to adsorb gas molecules at varying energy sites. Additionally, the amount of CO_2_ sorbed increased in the range of 0.02–0.1 mmol/g for the Toth model and 0.2–0.4 mmol/g for the Sips model, which implies that the spent shale has the potential to attain increased sorption capacity above the studied temperature and pressure. This further confirms the viability of spent shale absorbents.

The strong agreement with both models demonstrates the applicability of the Sips and Toth isotherms for describing the adsorption characteristics of pyrolysed materials, contrasting with the fits observed for the raw samples. This confirms the agreement of the spent shale supporting heterogeneous adsorption. The heterogeneity of the materials has been confirmed by the morphological display of the FESEM images.

Similarly, as shown in Table 3 and Table 4, the n-values of the Freundlich model are less than 1, which supports favorable and physical sorption. This is in agreement with the type of sorption that has been reported to be favorable for CO_2_ sequestration on several shale samples [50,51]. Finally, a monolayer form of sorption is confirmed by the Langmuir model.

### 2.4. Kinetic Studies

**Figure 11:**
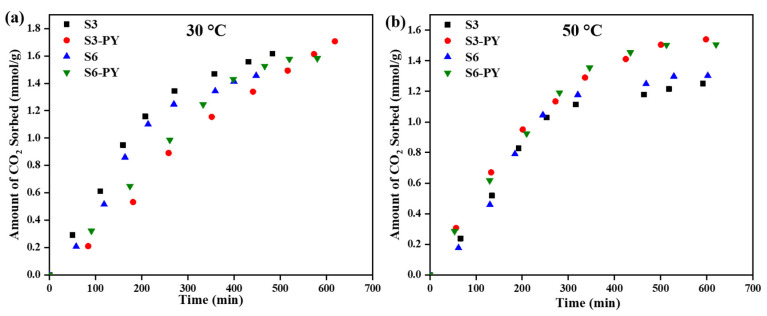
Rate of CO_2_ sorption on raw and pyrolyzed S3 and S6 at (**a**) 30 °C, (**b**) 50 °C.

The rate of sorption for the raw and pyrolyzed S3 and S6 at temperatures of 30 °C, 50 °C, and up to 8 MPa pressure is shown in Figure 11. The results revealed that the rates of sorption are reflections of the influence of the morphology, mineralogy, and the impact of pyrolysis. The information about the rate of adsorption was obtained from the curve fitting of the CO_2_ sorption results to the pseudo-first-order and pseudo-second-order models, with the parameters listed in Table 5 and Table 6. Thus far, the adsorption of CO_2_ on shale has been reported to be more diffusion-driven than chemisorption. However, there is a possibility of both scenarios occurring depending on the sorption behavior of the materials involved. These have been revealed from the sorption experimental results and the equilibrium model curves.

Based on the R^2^- values obtained for pseudo-first order and pseudo-second order being greater than 0.95 (see Table 5 and Table 6), it indicates that the rate of adsorption is driven by both diffusion (first-order) and chemical (second-order) processes. When this occurs, it means both models support the adsorption kinetics of the material, as pseudo-first order supports a diffusion phenomenon, while pseudo-second order supports a chemical phenomenon. This phenomenon is due to the minerals present in the shale samples, which contain some functional groups that can aid the chemical reaction of the shale with CO_2_ [52], along with the physisorption through the porous media. The models further validate the enhancement of the CO_2_ sorption capacity of shale by the pyrolysis method.

Table 7 presents a comparative analysis of the CO_2_ adsorption performance of the studied spent shale with other geological materials tested under similar pressure conditions (6–9 MPa) but at varying temperatures. The results indicate that the spent shale generally exhibits higher CO_2_ uptake than raw shale and coal samples evaluated at comparable pressures and slightly different thermal conditions. In some instances, its adsorption capacity was comparable to that of the reference materials; however, the superior performance of the spent shale can be attributed to the structural and surface modifications induced by pyrolysis. The high-temperature treatment enhanced pore development exposed additional active sites, and increased surface heterogeneity, all of which contributed to greater CO_2_ affinity. These findings demonstrate that thermal activation through pyrolysis significantly improves the sorption potential of shale for high-pressure CO_2_ storage applications.

Beyond adsorption capacity, spent shale offers several practical advantages over conventional adsorbents. As a by-product of oil shale processing, it is readily available at low or negligible cost, providing a highly cost-effective option for large-scale CO_2_ capture. Its mineral-rich framework imparts good mechanical strength, reducing the risk of structural collapse under high-pressure storage conditions. In addition, the predominantly inorganic composition enhances hydrothermal stability, ensuring that adsorption performance is maintained even under moist or thermally fluctuating environments. These attributes make spent shale not only a technically viable adsorbent but also a sustainable and scalable material for carbon capture applications.

## 3. Materials and Methods

### 3.1. Materials

Two Devonian shale samples were selected from the Marcellus Formation, USA, labeled S3 and S6 with high Total Organic Content (TOC) of 16.2% and 17.9%, respectively. Nitrogen (N_2_) gas was utilized for the regeneration, purging, and pyrolysis processes, while high-purity CO_2_ and helium (He) gases were used for the sorption process. A mortar grinder for sample pulverization and a heating tube furnace for the pyrolysis procedure were used respectively.

### 3.2. Sample Preparation

The shale samples were first cleaned and oven-dried at 100 °C for 4 h to remove volatile impurities. The dried material was then crushed into a fine powder using a mortar grinder and sieved to obtain particles within the 0.118–0.250 mm range. To preserve consistency prior to pyrolysis and adsorption measurements, the sieved samples were stored in airtight bags under controlled conditions. The pyrolysis of the powdered samples was carried out in a tube furnace (PROTHERM 5) using nitrogen gas (99.995% purity). The equipment set-up gradually increases the temperature from room temperature to 800 °C and holds for 4 h with a 5 °C/min ramping rate and 2 mL/min gas purging, then automatically cools to room temperature after the pyrolysis is completed. The pyrolysis temperature of 800 °C was selected based on reported studies that indicated that shale heated to ~800 °C undergoes extensive volatile escape, carbonate decomposition, and pore structure development [30,45], hence, resulting in enhanced adsorption capacity compared to lower temperatures. The char generated after the pyrolysis is referred to as a spent shale, which was used in this study for structural analysis and adsorption capacity measurement. These char samples were labeled S3-PY and S6-PY, respectively.

### 3.3. Sample Characterization

The porous classification of the raw and spent shale samples was examined through the BET/N_2_ adsorption/desorption techniques using the ASAP 2020 equipment (Micromeritics, Norcross, GA, USA) at the boiling point of N_2_ (−195.8 °C). The degassing of the samples was performed in a vacuum at 90 °C for an hour, followed by the injection of helium at 150 °C for 2 h to desorb the N_2_. The BET surface area was obtained from the BET multipoint data. Also, the amount of N_2_ sorbed at the relative pressure (P/P_0_ ≈ 1) was calculated, and the distribution of pore sizes and volume was determined through the Barrett-Joyner-Halenda (BJH) method.

Crystallographic phase identification and morphological structures of the samples were measured under XRD using the Malvern Panalytical equipment (Malvern, UK, with Cu Kα (λ = 0.1540 nm) radiation, ranging 2–70° 2θ angle, and a SUPRA 55VP FESEM (Field Emission Scanning Electron Microscopy, ZEISS, Oberkochen, Germany), respectively, before and after pyrolysis. Additional identification of the elements present in the raw and spent shale was examined through Energy-dispersive X-ray Spectroscopy (EDX) attached to the FESEM equipment. The nature of functional groups on the surface of the materials was determined through Fourier Transform Infrared (FTIR) Spectra using a Perkin Elmer spectrometer (Model: Frontier 01, Shelton, CT, USA) within the range of 4000–500 cm^−1^.

The Temperature Programmed Desorption (TPD) technique was utilized under the TPDRO model 1100 equipment (Thermo Fisher Scientific, Waltham, MA, USA) to measure the acidic/basic strength of the spent shale for sorption by identifying and characterizing the active sites present on the surface under the influence of temperature. Samples were heated under N_2_ gas at 28 °C, followed by the injection of CO_2_ until the temperature reached 75 °C, and held for 30 min to obtain the sorption measurement. Then TPD analysis was completed by injecting the Helium gas up to 500 °C for the next 30 min to desorb any form of chemisorbed CO_2_. The TPD results provide qualitative and semi-quantitative insights into the binding behavior of CO_2_ on shale surfaces.

### 3.4. Sorption Measurement

The volumetric sorption technique is one of the most prominent methods for evaluating the sorption capacity of a material. In this study, CO_2_ sorption measurements were performed at temperatures of 30 °C and 50 °C, and pressures of up to 8 MPa. The approach is to simulate a reservoir condition. The pressure was increased by 1 MPa per section to obtain an equilibrium until the maximum pressure was reached. The amount of gas sorbed at each section was calculated using the ideal gas law with a substantial compressibility factor. The Peng-Robinson Equation of State was further employed to determine the compressibility factors at interval pressures.

(1) $$P_{g}=P_{i}-P_{f}$$

(2) $$n_{g}=\frac{V_{g}P_{g}}{Z_{g}RT}$$

(3) $$n_{g}=\frac{V_{g}}{RT}\left(\frac{P_{i}}{Z_{i}}\right.-\left.\frac{P_{f}}{Z_{f}}\right)$$

Equation (1) is an expression for the equilibrium pressure attained at each section, where *P_i_* and *P_f_* are the initial gas pressure injected into the sorbent and the final pressure obtained at equilibrium, respectively. The amount of gas sorbed at each section is expressed by Equations (2) and (3), where *Z* is the compressibility factor measured by the initial and final pressure at each section. R denotes the gas constant, and T refers to temperature, which is also constant throughout a certain section [59]. At 50 °C and pressures above 73.8 bar, CO_2_ is in the supercritical state; thus, the isotherm data represent supercritical adsorption behavior. The experimental apparatus used for these measurements has been previously validated for high-pressure and supercritical CO_2_ adsorption studies, ensuring accuracy and reliability. These measurements provide quantitative adsorption capacities relevant for geological storage applications.

### 3.5. Isotherm Models

Some two- and three-parameter isotherm models were employed to validate the experimental results of this study. The equilibrium parameters essential to explain the sorption process were obtained using the non-linear regression (OriginPro 2022). This helps to estimate the values of the parameters by minimizing the sum of squared residuals, and the quality of the fit was assessed based on the coefficient of determination (R^2^) and the root mean square error (RMSE). This approach ensures robust and reproducible parameter estimation.

#### 3.5.1. Langmuir Model

This is one of the most common isotherm models employed for gas-solids sorption. It assumes that both sorbate and sorbent behave in an ideal manner at isothermal conditions, whereby the sorbates are distributed homogeneously on the surface of a sorbent, thereby forming a monolayer type of sorption [60,61]. It also presumes an equilibrium between the rate at which gas sorbs on the surface and the rate at which the solid desorbs the gas afterward [38,62]. The equilibrium Langmuir isotherm can be expressed as:

(4) $$q_{e}=\frac{q_{m}k_{L}P}{1+k_{L}P}$$

The *q_m_* and *k_L_* are the two parameters of the Langmuir model, where *q_m_* denotes the maximum sorption capacity, while k_L_ is the constant.

#### 3.5.2. Freundlich Model

The Freundlich model is one of the oldest known empirical models with two parameters, frequently employed for the sorption of gases [63]. It assumes the sorbates and sorbent interaction are in a heterogeneous system where the sorption is not limited to a monolayer formation, i.e., a formation of multilayer sorption is possible [64]. It best describes a non-ideal and reversible type of sorption. In addition, it describes the active site distribution, surface heterogeneity, and the energy of sorption of each site [65,66]. It can be expressed in a non-linear form in Equation (5), whereby the adsorption intensity (1/*n_F_*) represents the energy distribution and measures the heterogeneity of adsorbent sites. At 0 < 1/*n_F_* < 1, sorption is considered favorable, and when 1/*n_F_* > 1, the sorption process is unfavorable, and irreversible when = 1. While *k_F_* is the sorption potential of a sorbent.

(5) qe=kFP1nF

#### 3.5.3. Sips Model

The Sips isotherm model is an empirical three-parameter model also known as a hybrid isotherm of the Langmuir and the Freundlich models [67]. This isotherm model is presented to better predict the heterogeneity of the sorption surfaces and to overcome the inadequacies of the Freundlich isotherm’s increasing sorbate concentration [68]. It tends toward the Freundlich isotherm when the concentration of the sorbate is low and tends to predict the Langmuir isotherm when the concentration is high, forming a monolayer sorption phenomenon [69]. Equation (6) expresses the Sips model form.

(6) qe=qmKSP1nS1+KSP1nS

The maximum sorption capacity, *q_m_*, constant, *K_S_*_,_ and the Sips exponential, 1/*n_S_*_,_ are the three parameters of the model.

#### 3.5.4. Toth Model

The Toth model is an empirical isotherm model designed to improve the fitness of the Langmuir model to experimental results at relatively high pressures. It describes heterogeneous sorption systems, and it is appropriate for both low and high pressures or concentration ranges [60,70]. It describes a variety of systems with consistent sub-monolayer coverage, with its equilibrium expression linearized in Equation (7).

(7) qe=qmKTP1+KTPnT1nT

The *q_m_*, *K_T_*_,_ and *n_T_* represent the maximum sorption capacity, the Toth model constant, and the Toth isotherm exponential, respectively. Where the Toth exponential, *n_T_*, denotes the surface heterogeneity, usually less than or equal to unity (0 < 1/*n_T_* < 1).

### 3.6. Kinetic Model

This model describes the sorption mechanism of an adsorbate molecule on the surface of a sorbent under the influence of the sorption equilibrium time [71]. The kinetic study is essential for the sorption process because it evaluates the sorption capacity of the sorbent, the total mass transfer of the sorbate on the sorbent, the pathway of the reaction, and the sorption mechanism [72]. In this study, the pseudo-first-order and pseudo-second-order reactions were employed to best explain the CO_2_ sorption mechanism and equilibrium rate in correlation with the studied data.

#### 3.6.1. Pseudo-First-Order Model

This is the first-order rate of adsorption suggested by Lagergren [73] to describe a liquid-solid sorption phase [74]. It assumes that the rate of sorption of a sorbate is an expression of the difference between the saturation concentration and the amount of uptake with time. This model is expressed in Equation (8).

(8) $$q_{t}=q_{e}\left(1-e^{-k_{1}\;t}\right)$$

*q_t_* and *q_e_* denote the uptake at a time “*t*” and equilibrium, respectively, and *k*_1_ is the rate constant.

#### 3.6.2. Pseudo-Second-Order Model

The pseudo-second order, on the other hand, assumes that the kinetic rate of sorption could not be limited to chemisorption, i.e., a covalent bond or electrons shared between the sorbate and the sorbent [74]. It applies to all sorption processes that involve internal particle diffusion and external film diffusion, where the total rate of adsorption is assumed [75]. Equation (9) best expresses the pseudo-second order

(9) $$q_{t}=\frac{q_{e}^{2}k_{2}t}{1+q_{e}k_{2}t}\;$$

*q_t_* and *q_e_* denote the uptake at a time “*t*” and equilibrium, respectively, while *k*_2_ is the rate constant.

## 4. Conclusions

This study demonstrates the potential of pyrolyzed shale as an effective solid sorbent for CO_2_ capture. Thermal treatment at 800 °C under a nitrogen atmospheric condition significantly enhanced the shale’s physicochemical properties, including surface area and pore structure, which are critical for adsorption performance. Mineralogical analysis revealed the presence of quartz, feldspars, clays, and carbonate minerals, while TPD analysis confirmed the availability of active sites conducive to CO_2_ sorption. The spent shale achieved a notable CO_2_ sorption capacity of 1.62 mmol/g, outperforming several commercial sorbents. Adsorption isotherm modeling, particularly using the Sips and Toth models, indicated multilayer and heterogeneous adsorption behavior, while kinetic studies revealed that both diffusion and chemisorption processes governed the sorption mechanism. These findings position spent shale as a promising, low-cost, and sustainable sorbent for CO_2_ capture and sequestration. Beyond its technical viability, the reuse of spent shale for environmental remediation supports circular economy principles and resource valorisation. The selectivity of pyrolyzed shale toward CO_2_ under mixed-gas conditions has not yet been addressed in this study, owing to the fact that in actual flue gas, CO_2_ competes with N_2_, O_2_, and water vapor.

Beyond laboratory-scale adsorption, challenges remain in shaping, mechanical strength, and regeneration energy requirements that influence industrial viability. Future research should focus on process optimization, regeneration performance, and scale-up potential to further establish spent shale as a viable option for industrial carbon management applications.

## Figures and Tables

**Table 1:** The BET characterization values of the raw and pyrolyzed samples.

Samples	Pore Volume (cm^3^g^−1^)	Average Pore Size (nm)	Surface Area (m^2^g^−1^)
S3	0.014	3.1	31
S3-PY	0.023	9.4	33
S6	0.015	2.8	30
S6-PY	0.033	3.5	31

**Table 2:** Elemental composition (wt.%) of raw and pyrolyzed S3 and S6 obtained from FESEM-EDX.

Samples	C	O	Si	Al	Ca	Mg	K	Fe	S
S3	36.57	42.98	16.51	0.70	1.26	0.19	0.29	1.14	0.37
S3-PY	36.45	31.25	12.28	4.40	9.63	0.44	2.57	0.53	2.47
S6	33.78	31.43	16.07	5.50	4.06	0.60	3.17	2.77	2.27
S6-PY	15.67	42.42	12.43	2.35	24.10	0.51	1.25	0.32	0.94

**Table 3:** Isotherm model parameters’ values obtained at 30 °C.

30 °C					
Samples	Parameters	Langmuir	Freundlich	Sips	Toth
S3	q_m_ (mmol/g)	2.328 ± 0.022	-	1.487 ± 0.040	1.332 ± 0.031
	K (1/MPa)	0.182 ± 0.042	0.409 ± 0.021	0.186 ± 0.025	0.217 ± 0.057
	N	-	0.601 ± 0.095	1.821 ±0.031	0.231 ± 0.002
	R^2^	0.984	0.963	0.999	0.999
	RMSE	0.0042	0.0097	0.0004	0.0005
S3-PY	q_m_ (mmol/g)	3.339 ± 0.035	-	2.046 ± 0.063	1.724 ± 0.057
	K (1/MPa)	0.126 ± 0.022	0.423 ± 0.013	0.153 ± 0.005	0.183 ± 0.004
	N	-	0.676 ± 0.046	1.557 ± 0.027	0.288 ± 0.054
	R^2^	0.992	0.980	0.999	0.999
	RMSE	0.0029	0.0076	0.0002	0.0003
S6	q_m_ (mmol/g)	3.147 ± 0.057	-	1.659 ± 0.037	1.467 ± 0.056
	K (1/MPa)	0.119 ± 0.036	0.372 ± 0.018	0.122 ± 0.007	0.189 ± 0.007
	N	-	0.696 ± 0.057	1.980 ± 0.022	0.181 ± 0.009
	R^2^	0.981	0.965	0.999	0.996
	RMSE	0.0042	0.0011	0.0002	0.0015
S6-PY	q_m_ (mmol/g)	2.995 ± 0.038	-	1.882 ± 0.099	1.606 ± 0.009
	K (1/MPa)	0.161 ± 0.038	0.475 ± 0.064	0.171 ± 0.017	0.205 ± 0.001
	N	-	0.625 ± 0.019	1.743 ± 0.055	0.181 ± 0.036
	R^2^	0.984	0.966	0.997	0.999
	RMSE	0.0062	0.0013	0.0014	0.0006

**Table 4:** Isotherm model parameters’ values obtained at 50 °C.

50 °C					
Samples	Parameters	Langmuir	Freundlich	Sips	Toth
S3	q_m_ (mmol/g)	2.218 ± 0.029	-	1.382 ± 0.04	1.244 ± 0.03
	K (1/MPa)	0.183 ± 0.047	0.390 ± 0.023	0.174 ± 0.017	0.219 ± 0.006
	N	-	0.602 ± 0.057	1.955 ± 0.038	0.192 ± 0.004
	R^2^	0.979	0.967	0.998	0.998
	RMSE	0.0050	0.0100	0.0005	0.0006
S3-PY	q_m_ (mmol/g)	2.835 ± 0.021	-	1.944 ± 0.046	1.720 ± 0.074
	K (1/MPa)	0.160 ± 0.022	0.449 ± 0.044	0.189 ± 0.005	0.198 ± 0.005
	N	-	0.624 ± 0.014	1.494 ± 0.022	0.395 ± 0.038
	R^2^	0.995	0.981	0.999	0.999
	RMSE	0.0019	0.0065	0.0001	0.0003
S6	q_m_ (mmol/g)	2.792 ± 0.062	-	1.437 ± 0.043	1.299 ± 0.039
	K (1/MPa)	0.127 ± 0.048	0.348 ± 0.064	0.103 ± 0.013	0.196 ± 0.008
	N	-	0.687 ± 0.022	2.302 ± 0.032	0.116 ± 0.012
	R^2^	0.969	0.968	0.998	0.994
	RMSE	0.0091	0.0150	0.0007	0.0019
S6-PY	q_m_ (mmol/g)	2.910 ± 0.039	-	1.776 ± 0.083	1.526 ± 0.013
	K (1/MPa)	0.155 ±0.038	0.443 ± 0.062	0.162 ± 0.016	0.202 ± 0.002
	N	-	0.635 ± 0.020	1.801 ± 0.049	0.170 ± 0.063
	R^2^	0.983	0.964	0.997	0.999
	RMSE	0.0062	0.0129	0.0011	0.0001

**Table 5:** Kinetic model parameters’ values obtained at 30 °C.

30 °C					
Models	Parameters	S3	S3-PY	S6	S6-PY
Pseudo-First Order	K_1_ (min^−1^)	0.003 ± 0.001	0.002 ± 0.001	0.003 ± 0.001	0.003 ± 0.001
	q_e_ (mmol/g)	1.173± 0.010	1.1214 ± 0.066	1.9211 ± 0.010	1.965 ± 0.099
	R^2^	0.973	0.987	0.9755	0.986
	RMSE	0.0031	0.0011	0.0029	0.0023
Pseudo-Second Order	K_2_ (min^−1^)	0.001 ± 0.002	0.001 ± 0.001	0.001 ± 0.0004	0.001 ± 0.0001
	q_e_ (mmol/g)	3.484 ± 0.053	4.103 ± 0.046	3.037 ± 0.062	2.945 ± 0.074
	R^2^	0.982	0.995	0.962	0.962
	RMSE	0.0070	0.0016	0.0094	0.0069

**Table 6:** Kinetic model parameters’ values obtained at 50 °C.

50 °C					
Models	Parameters	S3	S3-PY	S6	S6-PY
Pseudo-First Order	K_1_ (min^−1^)	0.002 ± 0.001	0.002 ± 0.001	0.003 ± 0.001	0.002 ± 0.001
	q_e_ (mmol/g)	0.925 ± 0.012	0.961 ± 0.011	1.671 ± 0.012	2.251 ± 0.012
	R^2^	0.9607	0.989	0.973	0.983
	RMSE	0.0040	0.0034	0.0045	0.0042
Pseudo-Second Order	K_2_ (min^−1^)	0.002 ± 0.001	0.001 ± 0.002	0.001 ± 0.001	0.0004 ± 0.0003
	q_e_ (mmol/g)	1.939 ± 0.024	2.494 ± 0.013	2.582 ± 0.036	3.692 ± 0.023
	R^2^	0.962	0.995	0.967	0.981
	RMSE	0.0081	0.0015	0.0012	0.0048

**Table 7:** Comparison of the sorption capacity of the shale sample within 6–8 MPa at different temperatures.

Adsorbents	CO_2_ Sorption Capacity (mmol/g)	Temperature (°C)	Pressure (MPa)	References
Shale	0.25	40	8	Hu et al. [53]
Shale	0.20	60	8	Hu et al. [53]
Shale	0.15	45	8.5	Khosrokhavar et al. [54]
Shale	0.18	45	8.5	Khosrokhavar et al. [54]
Shale	1.10	25	8	Abdulkareem et al. [38]
Coal	0.81	35	6.9	Weniger et al. [55]
Coal	0.64	35	7.9	Weniger et al. [55]
Coal	2.20	40	8	Han et al. [56]
Coal	1.91	45	8.1	de Oliveira et al. [57]
Coal	1.30	27	6	Abunowara et al. [58]
Spent shale	1.62	30	8	This work

## Data Availability

Data will be made available on request.

## Abbreviations

The following abbreviations are used in this manuscript:

BET	Brunauer–Emmett–Teller
BJH	Barrett-Joyner-Halenda
CCUS	Carbon Capture Uptake and Sequestration
EDX	Energy-dispersive X-ray Spectroscopy
EoS	Equation of State
FESEM	Field Emission Scanning Electron Microscopy
FTIR	Fourier Transform Infrared
GHG	Greenhouse Gases
IUPAC	International Union of Pure and Applied Chemistry
MPa	MegaPascal
SDG	Sustainable Development Goal
TOC	Total Organic Content
TPD	Temperature-Programmed Desorption
UN	United Nation
XRD	X-ray Diffraction
RMSE	Root Mean Square Error
